# High Blood Uric Acid Is Associated With Reduced Risks of Mild Cognitive Impairment Among Older Adults in China: A 9-Year Prospective Cohort Study

**DOI:** 10.3389/fnagi.2021.747686

**Published:** 2021-10-14

**Authors:** Chen Chen, Xueqin Li, Yuebin Lv, Zhaoxue Yin, Feng Zhao, Yingchun Liu, Chengcheng Li, Saisai Ji, Jinhui Zhou, Yuan Wei, Xingqi Cao, Jiaonan Wang, Heng Gu, Feng Lu, Zuyun Liu, Xiaoming Shi

**Affiliations:** ^1^China CDC Key Laboratory of Environment and Population Health, National Institute of Environmental Health, Chinese Center for Disease Control and Prevention, Beijing, China; ^2^Department of Big Data in Health Science, School of Public Health, Zhejiang University School of Medicine, Hangzhou, China; ^3^Division of Non-communicable Disease and Healthy Ageing Management, Chinese Center for Disease Control and Prevention, Beijing, China; ^4^Center for Global Health, School of Public Health, Nanjing Medical University, Nanjing, China; ^5^Beijing Municipal Health Commission Information Center, Beijing Municipal Health Commission Policy Research Center, Beijing, China; ^6^Center for Clinical Big Data and Analytics, Second Affiliated Hospital, Zhejiang University School of Medicine, Hangzhou, China

**Keywords:** uric acid, mild cognitive impairment, cognitive function, older adults, prospective cohort study

## Abstract

**Background:** It remains unsolved that whether blood uric acid (UA) is a neuroprotective or neurotoxic agent. This study aimed to evaluate the longitudinal association of blood UA with mild cognitive impairment (MCI) among older adults in China.

**Methods:** A total of 3,103 older adults (aged 65+ years) free of MCI at baseline were included from the Healthy Aging and Biomarkers Cohort Study (HABCS). Blood UA level was determined by the uricase colorimetry assay and analyzed as both continuous and categorical (by quartile) variables. Global cognition was assessed using the Mini-Mental State Examination four times between 2008 and 2017, with a score below 24 being considered as MCI. Cox proportional hazards models were used to examine the associations.

**Results:** During a 9-year follow-up, 486 (15.7%) participants developed MCI. After adjustment for all covariates, higher UA had a dose-response association with a lower risk of MCI (all *P*_*for  trend*_ < 0.05). Participants in the highest UA quartile group had a reduced risk [hazard ratio (HR), 0.73; 95% (CI): 0.55–0.96] of MCI, compared with those in the lowest quartile group. The associations were still robust even when considering death as a competing risk. Subgroup analyses revealed that these associations were statistically significant in younger older adults (65–79 years) and those without hyperuricemia. Similar significant associations were observed when treating UA as a continuous variable.

**Conclusions:** High blood UA level is associated with reduced risks of MCI among Chinese older adults, highlighting the potential of managing UA in daily life for maintaining late-life cognition.

## Introduction

Uric acid (UA) is a chemical which is created when the human body breaks down purines and then catalyzed by xanthine oxidase. Blood UA level is determined by the residual quantities between dietary purine intake and renal excretion. Hyperuricemia, the status of an abnormally high level of UA in the blood, is a prerequisite for gout (Zhu et al., 2011). Both hyperuricemia and gout confer a high risk for subsequent metabolic syndrome (Choi et al., 2007) and cardiovascular diseases (CVDs) (Feig et al., 2008; Richette et al., 2014). However, as a natural antioxidant, the antioxidant properties of UA may protect against the detrimental effect of oxidative stress among people with central nervous system disorders (Euser et al., 2009; Gong et al., 2012). Some debates have been raised that urate-lowering-therapies might paradoxically expose patients to a high risk of neurodegenerative disease (such as dementia) (Khan et al., 2016; Singh and Cleveland, 2018b).

To date, many studies have explored the associations between blood UA levels and cognition or dementia in developed countries, but the results remain in dispute. Positive (De Vera et al., 2008; Euser et al., 2009; Irizarry et al., 2009; Cicero et al., 2015; Lu et al., 2016; Pellecchia et al., 2016; Ye et al., 2016; Tuven et al., 2017), negative (Latourte et al., 2018; Singh and Cleveland, 2018a; Alam et al., 2020), U-shaped (Huang et al., 2019), or no associations (Iuliano et al., 2010) of blood UA levels with cognition have been reported. In China, some cross-sectional studies support the neuroprotective role of UA in cognition. However, most of them were limited to their cross-sectional nature and the relatively small sample size (Li et al., 2010; Wu et al., 2013; Liu et al., 2017; Wang F. et al., 2017; Xiu et al., 2017; Xu et al., 2017). To our best knowledge, only three relevant longitudinal studies have been conducted, with one on Parkinson’s disease (PD) (Hu et al., 2020) or one on dementia (Hong et al., 2015). The only one on cognition was limited to its relatively short follow-up time (ranged from 1.3 to 2.4 years) (Wang T. et al., 2017) (For detailed information see Supplementary Table 1). Therefore, investigations on the long-term effect of UA on cognition in general Chinese populations are urgently needed.

Therefore, based on 9-year follow-up data from a community-based multi-wave cohort of older adults (65 years and older), the Healthy Aging and Biomarkers Cohort Study (HABCS), this study aimed to evaluate the longitudinal associations of blood UA levels with the risks of incident mild cognitive impairment (MCI) in general Chinese older population.

## Materials and Methods

### Study Population

**FIGURE 1:**
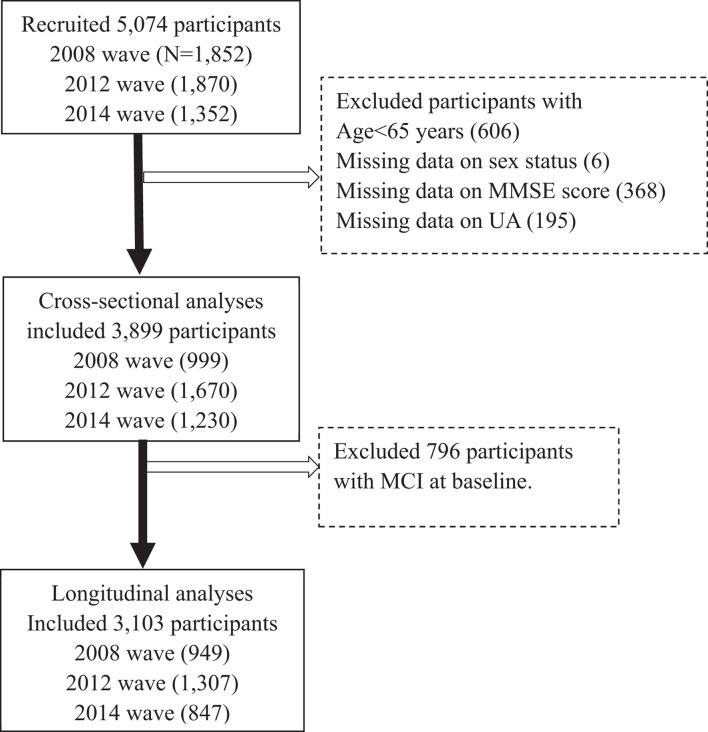
Flowchart of study participant enrollment in this study. MMSE, Mini-Mental State Examination. UA, uric acid.

The HABCS was launched in eight longevity areas in China in 2008, collecting comprehensive data, such as health status, and behavioral, laboratory, and anthropometric measurements. The follow-up surveys were conducted in 2012, 2014, and 2017. Due to the high death rate in older adults, new participants were recruited in the follow-up surveys to maintain a stable sample size for the dynamic cohort. Details of the survey design have been described elsewhere (Lv et al., 2019). A total of 5,074 older adults from the 2008 survey and the recruits from the follow-up surveys (2012–2014 waves) from the HABCS were included in this study. We excluded 606 participants aged < 65 years, six participants with missing data on sex, 368 participants on Mini-Mental State Examination (MMSE) score, and 195 participants on UA levels, and then excluded 796 participants with prevalent CI at baseline, leaving 3,103 participants free of CI for the final longitudinal analysis (Figure 1). All participants or their legal representatives gave written informed consent to participate in the baseline and follow-up surveys. The HABCS was approved by the Ethics Committee of Peking University and Duke University.

### Assessment of Blood Uric Acid Levels

Fasting venous blood was collected from all participants at the baseline wave (the year 2008–2014). The plasma was separated and stored at −20°C and delivered to the laboratory at Capital Medical University in Beijing for unified detection. Blood UA level was determined by uricase colorimetry assay. Details on quality control and assessments in the laboratory were described elsewhere (Yi et al., 2012). Blood UA levels > 420 μmol/L in men and UA levels > 360 μmol/L in women were defined as hyperuricemia (Zhang et al., 2011), which indicates above the UA normal range. Both continuous (i.e., per 10 μmol/L blood UA increment) and categorical (i.e., sex-specific quartiles of UA) type were constructed and used in all analyses.

### Assessment of Cognition

Cognition was assessed in the years 2008, 2012, 2014, and 2017, using the modified version of the widely used MMSE questionnaire. The MMSE included six cognitive domains (orientation, working memory, concentration, memory recall, language, and visuospatial ability), with a total score ranging from 0 to 30, and the higher score indicates better cognition. In HABCS, professionally trained staff interviewed participants at the baseline survey and subsequent follow-up surveys face-to-face at their homes. We asked the trained staff to review the MMSE form before each formal interview. Trained staff asked questions in order which were listed on the form, then scored immediately and documented score in the appropriate location. For participants who were unable to complete a question, we did not ask questions again (Katzman et al., 1988; Lv et al., 2019). We categorized cognition into two levels: no MCI (24 ≤ MMSE ≤ 30) and MCI (MMSE < 24) as done previously (Creavin et al., 2016; Yuan et al., 2019). We set MCI as the outcome and calculated the follow-up time for each participant from baseline to either the date of incident MCI, loss to follow-up (including death), or the end of the study period, whichever came first.

### Covariates

Age, sex, education, current marital status, current smoking, current alcohol consuming, regular exercise, and adequate medical service were considered. Education was categorized as having more than 1 year of schooling or no formal education. Current marital status was categorized as currently married or others. Current smoking and current alcohol consumption were categorized as with or without status. Regular exercise was classified into “yes” or “no” by the question “Do you do exercises regularly at present, including walking, playing ball, running, and Qigong?” Hypertension was defined as yes if a participant had an SBP ≥ 140 mmHg and/or a DBP ≥ 90, or self-reported suffering from hypertension. Diabetes mellitus was defined as yes if a participant had fasting glucose ≥ 7.0 mmol/L or self-reported current diabetes medication use. Self-reported history of heart disease, stroke and CVD were also considered. Standardized protocols were used to collect measurements of weight, height, and waist circumference (WC) (Lv et al., 2018). Central obesity was defined as 85 cm or larger of WC in men and 80 cm or larger of WC in women. These covariates have been demonstrated as important correlations with blood UA level (Norris et al., 2018), or cognition (Liu et al., 2018), or both (Lv et al., 2019).

### Statistical Analyses

Descriptive statistics of the baseline characteristics were presented as means ± standard deviation (SD) or percentages among the full sample and by the sex-specific UA quartile groups.

First, we evaluated the associations and dose-response relationship between blood UA and baseline cognition score, to confirm the findings from previous studies using a cross-section design.

Second, we ran the main analysis (prospective analysis), i.e., examining the association between blood UA quartiles (the lowest UA quartile as a reference category) and the risk of MCI. Cox proportional hazards model was used and hazard ratios (HRs) and 95% confidence intervals (CIs) were documented. Three models were considered. In model 1, we included age, sex, and education. In model 2, we further included current smoking, current alcohol consuming, marital status, regular exercise, BMI, central obesity, and adequate medical service based on model 1. In model 3, we additionally adjusted for hypertension, diabetes mellitus, heart disease, and stroke and CVD based on model 2. Then, given the high proportion of death in older adults, we used Fine and Gray competing risk models (Fine and Gray, 1999) to re-examine the association between blood UA and risk of developing MCI, using the similar models as above. Additionally, we used restricted cubic splines (RCS) to flexibly model and visualize the dose-response association of blood UA levels with the risk of MCI with three sex-specific knots at the 5th, 50th, and 95th percentiles of blood UA distribution in model 1. We performed subgroup analyses of the association between blood UA and the risk of developing MCI by age (65–79 vs ≥ 80 years), sex (men vs women), and hyperuricemia (without hyperuricemia vs with hyperuricemia) using model 3. We added the interaction items (e.g., age group × UA quantiles) to determine whether the associations differed by the subgroup.

Third, as MCI is a disease state rather than an acute event, such as stroke, we also explored the longitudinal associations between baseline blood UA and the repeated MMSE scores at follow-up examinations using a mixed-effect linear model (Littell et al., 1998). We repeated the three steps of analyses above using blood UA as a continuous variable (i.e., per 10 μmol/L blood UA increment).

Two sensitivity analyses were conducted to verify the robustness of the above estimations. First, we compared the baseline characteristics of the included and excluded participants. Second, because we included new participants enrolled in the follow-up surveys, we also compared the baseline characteristics of study participants by different enrolled times. A *P*-value of less than 0.05 (two-tailed) was considered. All analyses were carried out in SAS version 9.4 (SAS Institute, Cary, NC, United States).

## Results

### Cross-Sectional Associations of Blood Uric Acid and Cognition

We included 3,899 participants for the cross-sectional analysis and their basic characteristics were presented in Supplementary Table 2. We observed that higher blood UA levels were associated with higher cognitive scores (β = 0.08, for per 10 μmol/L blood UA level increment, *P* < 0.010), after fully adjusting of covariates in model 3. Additionally, compared with the participants in the lowest quartile group, those in the highest quartile group had 1.41 points higher cognitive scores (Table 1). We found consistent results of higher UA levels associated with higher cognition scores using dose-response testing in the cross-sectional analysis (Supplementary Figure 1).

**TABLE 1:** Cross-sectional associations of blood UA levels with the cognitive score.

Blood UA (μmol/L)	Model 1	Model 2	Model 3
	β (SE)	β (SE)	β (SE)
**Continuous variable**			
Per 10 μmol/L increment	0.09 (0.01)**	0.08 (0.01)**	0.08 (0.01)**
**By quartiles^a^**			
Q_1_	Ref.	Ref.	Ref.
Q_2_	0.85 (0.35)	0.46 (0.35)	0.32 (0.35)
Q_3_	1.53 (0.35)**	1.44 (0.36)**	1.27 (0.36)**
Q_4_	1.90 (0.36)**	1.66 (0.36)**	1.41 (0.36)**
P for linear trend	<0.010	<0.010	<0.010

*UA, uric acid; SE, standard estimation.*

*Model 1 adjusted for age, sex, and education; model 2 additionally adjusted for drinking, smoking, marital status, regular exercise, body mass index, central obesity, adequate medical service; model 3 additionally adjusted for hypertension, diabetes mellitus, self-reported history of heart disease, and stroke and cardiovascular disease.*

*^*a*^The cutoff values were 214.2, 264.0, and 319.0 μmol/L for women, 252.0, 304.6, and 364.5 μmol/L for men.*

**P < 0.01.

### Main Analysis: Prospective

### Associations of Blood Uric Acid With the Incidence of Mild Cognitive Impairment

For prospective analyses, we included 3,103 participants. The mean (SD) age of 3,103 older adults was 85.1 (±11.7) years, and approximately 54% (*n* = 1,688) were women. Participants with higher blood UA were more likely to have traditional cardiovascular disease risk factors (drinking, central obesity, hypertension, and diabetes mellitus), while had a higher proportion of the assumed healthy behaviors (regular exercise) (all *P* < 0.050, Table 2).

**TABLE 2:** Baseline characteristics of study participants included in the main analysis, in total and by UA quartiles^a^.

Characteristics	Total (*N* = 3103)	Q_1_ (*N* = 773)	Q_2_ (*N* = 777)	Q_3_ (*N* = 776)	Q_4_ (*N* = 777)	*P* Value
Age, mean ± SD, years	85.1 ± 11.7	82.8 ± 12.2	84.8 ± 11.6	85.8 ± 11.4	86.8 ± 11.2	< 0.001
Sex, women (%)	1,688 (54.4)	422 (54.6)	421 (54.2)	424 (54.6)	421 (54.2)	0.996
More than 1 year of education, yes (%)	1,098 (36.3)	274 (36.2)	258 (33.9)	276 (36.6)	290 (38.6)	0.319
Currently married, yes (%)	1,170 (38.5)	318 (41.6)	294 (38.6)	292 (38.4)	266 (35.5)	0.113
Regular exercise, yes (%)	552 (18.4)	105 (13.9)	150 (19.7)	144 (19.0)	153 (20.8)	0.003
Current smoking, yes (%)	573 (18.8)	152 (19.9)	134 (17.4)	142 (18.5)	145 (19.3)	0.628
Current alcohol drinking, yes (%)	510 (16.8)	135 (17.6)	101 (13.3)	139 (18.2)	135 (18.0)	0.028
BMI, mean ± SD, kg/m^2^	21.5 ± 10.1	20.8 ± 3.4	21.9 ± 17.2	21.4 ± 6.3	21.8 ± 7.7	0.170
Central obesity ^b^, yes (%)	1,303 (42.3)	308 (40.1)	315 (41.0)	318 (41.3)	362 (46.8)	0.031
Adequate medical service, yes (%)	2,864 (93.9)	706 (92.4)	722 (94.1)	728 (94.9)	708 (94.1)	0.212
Hypertension, yes (%)	1,730 (55.8)	352 (45.6)	410 (52.9)	473 (61.0)	495 (63.8)	< 0.001
Diabetes mellitus, yes (%)	331 (10.7)	74 (9.6)	65 (8.4)	99 (12.8)	93 (12.0)	0.017
Heart Disease, yes (%)	232 (7.7)	58 (7.7)	46 (6.1)	61 (8.1)	67 (8.9)	0.212
Stroke/CVD, yes (%)	165 (5.4)	50 (6.6)	35 (4.6)	35 (4.6)	45 (5.9)	0.232

*UA, uric acid; SD, standard deviation; BMI, body mass index; CVD, cardiovascular disease.*

*Values are given as No. (%) unless otherwise stated.*

*^*a*^The cutoff values were 213.9, 265.0, and 320.9 μmol/L for women, and 252.0, 305.6, and 365.1 μmol/L for men.*

*^*b*^Central obesity was defined as waist circumference ≥ 80 cm in women and waist circumference ≥ 85 cm in men.*

**FIGURE 2:**
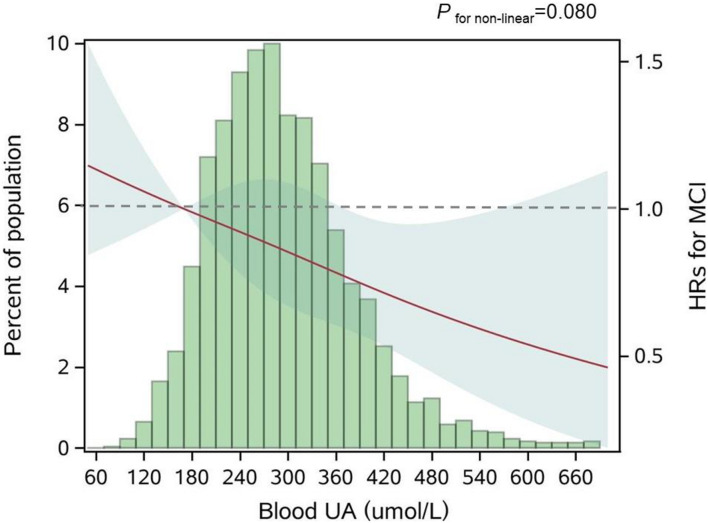
Adjusted dose-response association between blood UA and risk for MCI. UA, uric acid; HR, hazard ratio; MCI, mild cognitive impairment. Blood UA was coded using a restricted cubic spline (RCS) function with three sex-specific knots, which approximately corresponded to the 5th (170.0 μmol/L), 50th (281.6 μmol/L), and 95th (454.3 μmol/L) percentiles of blood UA distribution. The solid red line represents the adjusted hazard ratio for the risk of MCI for any value of UA compared to participants with 170.0 μmol/L (P5) of blood UA level, with light green shaded areas showing 95% confidence intervals derived from restricted cubic spline regressions. The green histograms show the fraction of the population with the different levels of blood UA. The dashed gray line refers to the reference for the association at an HR of 1.0. *P* for non-linear = 0.080.

**FIGURE 3:**
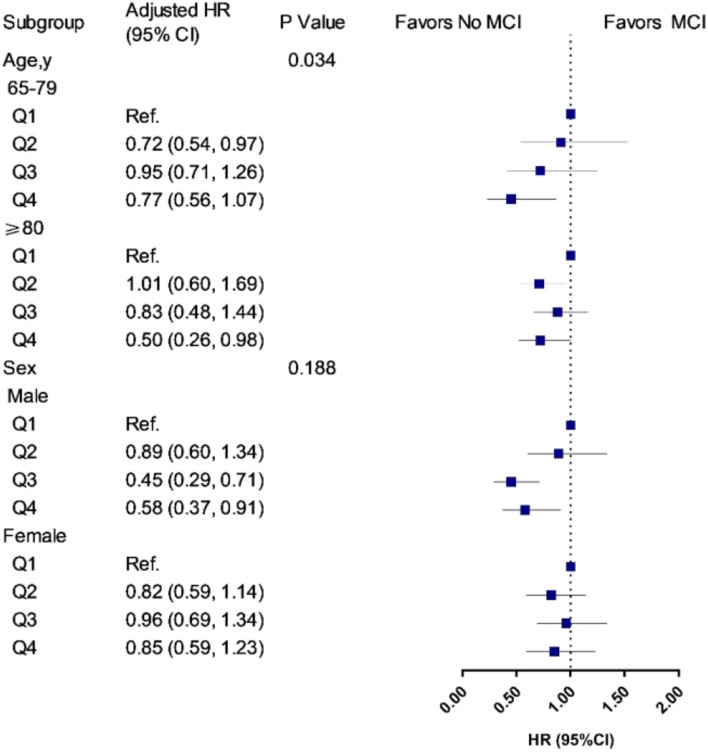
Associations of UA quartiles with the risk of MCI in age and sex subgroups. HR, hazard ratio; CIs, confidence intervals. The model was adjusted for age (not in age subgroup) and sex (not in sex subgroup), education, drinking, smoking, marital status, regular exercise, body mass index, central obesity, adequate medical service, hypertension, diabetes mellitus, self-reported history of heart disease, and stroke/CVD. Q1 was defined as the reference group. In the age subgroup, participants aged 65–79 years, the cutoff values of UA quartiles were 245.4, 291.6, and 355.4 μmol/L for men, and 195.2, 242.0, and 296.4 μmol/L for women; participants aged 80 years and older, the cutoff values of UA quartiles were 262.0, 315.0, and 377.1 μmol/L for men, and 220.0, 270.7, and 329.8 μmol/L for women. In the sex subgroup, the cutoff values were 252.0, 305.6, and 365.1 μmol/L for men, and 213.9, 265.0, and 320.9 μmol/L for women.

During a 9-year follow-up, 486 (15.7%) participants developed MCI. From three adjusted models in Table 3, we found that higher UA levels had a lower risk of developing MCI. After full adjustment of covariates in model 3, compared with participants in the lowest quartile group of UA level (Q1), those in the highest quartile group (Q4) had a 27% lower risk of MCI [HR_*Q4 vs Q1*_ = 0.73 (95% CIs: 0.55–0.96)]. The association was still robust after considering death as a competing risk [HR_*Q4 vs Q1*_ = 0.67 (95% CIs: 0.51–0.88)]. The dose-response relationship between blood UA and the risk of MCI was shown in Figure 2 (*P*_*for  non–linear*_ = 0.080). When treated UA as a continuous variable, we observed that per 10 μmol/L blood UA increment was associated with 2% decreased risk of incident MCI in competing risk model 3 [HR_*per 10*μ *mol/L*_ = 0.98 (95% CIs: 0.97–0.99)]. Subgroup analyses stratified by age and sex are shown in Figure 3. The associations were more pronounced in the younger older adults (65–79 years) (*P*_*for interaction*_ = 0.034). Compared with the lowest quartile group of UA level (Q1), those in the highest quartile group (Q4) had a 50% lower risk of developing MCI [HR_*Q4 vs Q1*_ = 0.50 (95% CI: 0.26–0.98)]. Partially due to the relatively small sample size, some of the associations of blood UA levels with the risk of MCI became non-significant (e.g., that in women). In addition, we observed that the association of blood UA on the risk of MCI remained statistically significant among those without hyperuricemia [HR_Q4 vs Q1_ = 0.64 (95% CI: 0.48–0.85)] (Supplementary Table 3). However, no significant associations were found for older adults with UA levels above the normal range (i.e., with hyperuricemia) [HR_*Q4 vs Q1*_ = 2.19 (0.81–5.88)].

**TABLE 3:** Longitudinal associations of blood UA levels with the risk of MCI.

Blood UA (μmol/L^a^)	Model 1	Model 2	Model 3
	HR (95% CI)	HR (95% CI)	HR (95% CI)
**Cox proportional hazards model**			
Per 10 μmol/L increment	0.99 (0.98, 0.10)	0.99 (0.98, 1.00)	0.99 (0.97, 0.10)
Q1	Ref.	Ref.	Ref.
Q2	0.84 (0.66, 1.06)	0.84 (0.66, 1.07)	0.82 (0.64, 1.06)
Q3	0.81 (0.64, 1.04)	0.83 (0.65, 1.06)	0.73 (0.56, 0.95)
Q4	0.74 (0.57, 0.96)	0.75 (0.57, 0.98)	0.73 (0.55, 0.96)
*P*_*for trend*_	0.022	0.036	0.013
**Fine and Gray competing risks model ^b^**			
Per 10 μmol/L increment	0.98 (0.97, 0.99)	0.98 (0.97, 0.99)	0.98 (0.97, 0.99)
Q1	Ref.	Ref.	Ref.
Q2	0.90 (0.71, 1.14)	0.89 (0.69, 1.14)	0.88 (0.68, 1.13)
Q3	0.89 (0.70, 1.13)	0.80 (0.62, 1.04)	0.79 (0.61, 1.02)
Q4	0.70 (0.54, 0.91)	0.67 (0.51, 0.88)	0.67 (0.51, 0.88)
*P*_*for trend*_	0.014	0.018	0.003

*UA, uric acid; HR, hazard, ratio; CI, confidence interval.*

*Model 1 adjusted for age, sex, and education; model 2 additionally adjusted for drinking, smoking, marital status, regular exercise, body mass index, central obesity, adequate medical service; model3 additionally adjusted for hypertension, diabetes mellitus, self-reported history of heart disease, and stroke and cardiovascular disease.*

*^*a*^The cutoff values were 213.9, 265.0, and 320.9 μmol/L for women, and 252.0, 305.6, and 365.1 μmol/L for men.*

*^*b*^Adjusted for death as competing risk.*

### Longitudinal Associations of Blood Uric Acid With Mini-Mental State Examination Score

We used the same dataset of 3,103 participants above to examine the longitudinal associations of blood UA with MMSE score. We found that higher blood UA was associated with a fewer decline in the MMSE score over time (Supplementary Table 4). The adjusted mean difference in annual cognitive decline rate, changed by the interaction of per 10 μmol/L increment and follow-up time, was −0.02 (0.005) points (*P* < 0.001).

### Sensitivity Analyses

In sensitivity analyses: (1) Differences in demographic characteristics were found between eligible and excluded participants. Those who were excluded were more likely to be younger and women, and had lower blood UA levels; and had more healthy characteristics (a higher proportion of education, and a lower proportion of smoking, central obesity, hypertension, and heart disease) (all *P* < 0.05, Supplementary Table 5). (2) Different characteristics of participants by enrolled time (i.e., 2008 wave, 2012 wave, and 2014 wave) were observed (Supplementary Table 6).

## Discussion

In this community-based longitudinal study of 3,103 Chinese older adults free of MCI at baseline, we found that higher UA was associated with a lower risk of developing MCI during the 9 years follow-up. Furthermore, these associations were generally stronger in the younger older adults (65–79 years) and those without hyperuricemia. The findings confirm the neuroprotective effect of UA in Chinese older populations.

The neuroprotective effects of blood UA we observed are generally consistent with that from the previous epidemiological studies. A recent cross-sectional study using data from Beijing Longitudinal Study on Aging II showed the beneficial role of high blood UA levels on CI (Xiu et al., 2017). A cohort study from Netherland, with over 11-year follow-up, found that higher serum UA levels at baseline predicted better cognition in later life (Euser et al., 2009). Similarly, another cohort (Lu et al., 2016) identified the potential neuroprotective role of UA on Alzheimer’s disease in a 5-year follow-up. However, there are still some studies that failed to observe the neuroprotective role of UA in dementia (Latourte et al., 2018; Singh and Cleveland, 2018a; Alam et al., 2020). The heterogeneity in study populations and outcome measurements might partially explain the different findings across studies.

Several possible biological mechanisms may elucidate the observed association of higher normal UA levels with a lower risk of MCI in the current study. First, in a urate oxidase mouse model with hemi-parkinsonism, genic mutated urate oxidase resulted in increased brain urate concentrations and substantially attenuated toxic effects of 6-hydroxydopamine on neurochemicals and rotational behavior. Additionally, transgenic urate oxidase decreased brain urate, which exacerbated neural functional lesions (Chen et al., 2013). Second, UA exerts its important antioxidant property in human bodies by acting as a direct scavenger of oxygen and hydroperoxyl radicals and forming stable complexes with iron ions (Davies et al., 1986), which further reduces the risk of MCI (Gackowski et al., 2008; Cervellati et al., 2013). Third, nutrition status might partly play a role in the UA-cognition relationship. Hypouricemia, a status of the abnormally low level of UA in the blood, is a well-established biomarker of poor nutritional status (Tseng et al., 2012). Meanwhile, malnutrition is a serious issue in older adults with MCI or dementia (Cederholm et al., 2014).

In our study, the association of blood UA with the risk of MCI appeared to be more pronounced in the younger older adults (65–79 years) and those without hyperuricemia. The age-specific protective effects of high blood UA on the risk of MCI might be explained by the fact that the younger older adults have a lower aging rate, and to be more sensitive to the beneficial effects of blood UA on cognition (Vauzour et al., 2017). Furthermore, survivor bias might exist in our study population. The unexpected non-significant result among those without hyperuricemia might be due to occasional chance and the relatively small sample size (N_*hyperuricemia*_ = 404). Besides, from the dose-response test (Figure 2), we noticed that the 95% CIs for the risk estimates became extremely wider when the UA levels were higher than about 420 μmol/L, which was around the cutoff value of the UA normal range (blood UA levels > 420 μmol/L in men and UA levels > 360 μmol/L in women were defined as hyperuricemia). These older adults with hyperuricemia might not share the same underlying etiology pathway relative to those without hyperuricemia.

The strengths of this study include its prospective cohort design and a relatively large sample size of older adults. However, several limitations should be considered. First, comprehensive neuropsychological assessments and the diagnoses of specific dementia or Parkinson’s disease were not available in our study, so we could not capture the detailed aspects and clinical stage of cognition. Nevertheless, (i) we repeated measured the MMSE four times, which would allow us to capture the global patterns of cognitive fluctuation over time; (ii) the MMSE was commonly used as part of the evaluation for possible dementia (Creavin et al., 2016). Second, the UA levels were measured only once, and may not reflect the true value and ignore the fluctuation. Third, residual confounding could not be ignored, such as, we did not collect information on dietary habits and medication, which might serve as confounding factors in the UA-cognition association. Due to these limitations, we need to be cautious when interpreting our results. Given that our study focused on the Chinese older adults, more studies are warranted to further investigate the associations in other ethnic groups.

## Conclusion

By evaluating the associations of blood UA with the risk of MCI, our findings suggest the protective role of high blood UA among Chinese older adults. The findings highlight the potential of managing UA in daily life for maintaining late-life cognition, with important clinical practice implications, and deserves further verifications.

## Data Availability Statement

The data that support the findings of this study are available from the corresponding author XS, upon reasonable request.

## Ethics Statement

The studies involving human participants were reviewed and approved by the Ethics Committee of Peking University and Duke University. The patients/participants provided their written informed consent to participate in this study.

## Author Contributions

ZL and XS: study concept and design. CC and XL: acquisition, analysis, interpretation of data, drafting of the manuscript, and statistical analysis. YLv, XC, ZL, and XS: critical revision of the manuscript for important intellectual content. ZL and XS: funding acquisition and supervision. ZY, FZ, YLi, CL, SJ, JZ, YW, XC, JW, HG, and FL: administrative, technical, or material support. All authors contributed to the article and approved the submitted version.

## Conflict of Interest

The authors declare that the research was conducted in the absence of any commercial or financial relationships that could be construed as a potential conflict of interest.

## Publisher’s Note

All claims expressed in this article are solely those of the authors and do not necessarily represent those of their affiliated organizations, or those of the publisher, the editors and the reviewers. Any product that may be evaluated in this article, or claim that may be made by its manufacturer, is not guaranteed or endorsed by the publisher.
